# The MicroRNA MiR-29c Alleviates Renal Fibrosis via TPM1-Mediated Suppression of the Wnt/β-Catenin Pathway

**DOI:** 10.3389/fphys.2020.00331

**Published:** 2020-04-14

**Authors:** Huiya Huang, Xiaozhong Huang, Shengnan Luo, Huidi Zhang, Feifei Hu, Ruyi Chen, Chaoxing Huang, Zhen Su

**Affiliations:** ^1^Department of Nephrology, The First Affiliated Hospital of Wenzhou Medical University, Wenzhou, China; ^2^Department of Intensive Care Unit, The First Affiliated Hospital of Wenzhou Medical University, Wenzhou, China; ^3^Department of Pediatric Surgery, The Second Affiliated Hospital and Yuying Children’s Hospital of Wenzhou Medical University, Wenzhou, China

**Keywords:** miR-29c, fibrosis, Wnt, β-catenin, TPM1

## Abstract

**Purpose:**

This study aimed to evaluate the mechanism by which miR-29c expression in fibroblasts regulates renal interstitial fibrosis.

**Methods:**

We stimulated NRK-49F cells with TGF-β1 to mimic the effects of fibrosis *in vitro*, while unilateral ureteral obstruction (UUO) was performed to obstruct the mid-ureter in mice. MiR-29c mimic or miR-29c inhibitor was used to mediate genes expressions *in vitro*. The recombinant adeno associated virus (rAAV) vectors carrying a FSP1 promoter that encodes miR-29c precursor or miR-29c inhibitor was used to mediate genes expressions *in vivo*, and a flank incision was made to expose the left kidney of each animal.

**Results:**

In the present study, TGF-β1 was demonstrated to regulate miR-29c expression through Wnt/β-catenin signaling. In contrast, miR-29c appears to inhibit the Wnt/β-catenin pathway by suppressing TPM1 expression. As suggested by this feedback mechanism, miR-29c may be a key fibrosis-related microRNA expressed by fibroblasts in TGF-β1/Wnt/β-catenin-driven renal fibrosis, and manipulation of miR-29c action may accordingly offer a potential therapeutic pathway for renal fibrosis treatment.

**Conclusion:**

MiR-29c expression was downregulated in UUO mouse kidneys as well as TGF-β1-treated NRK-49F cells, which thus inhibits myofibroblast formation via targeting of TPM1. Additionally, the production of extracellular matrix (ECM) in renal fibroblasts appears to be controlled by the reciprocal regulation of miR-29c action and the Wnt/β-catenin pathway.

## Introduction

Renal interstitial fibrosis – characterized by the accumulation of extracellular matrix (ECM), renal fibroblast and myofibroblast along with inflammation cell infiltration into renal tubular interstitial fluids and structural deterioration – often promotes the progression of chronic kidney disease (CKD) to its end-stage (Eddy, 1996; Satoh et al., 2001; Taneda et al., 2003). Fibroblasts are the main producers of interstitial ECM among all cell types (Nath, 1992; Grande and López-Novoa, 2009; Humphreys, 2018). Renal interstitial fibrogenesis is mainly driven by various profibrotic growth factors, which act to establish fibrogenic microenvironments within interstitial space. Because the expression of transforming growth factor (TGF)-β1 is upregulated in all types of CKD, in both experimental models and clinical settings, it is likely the most potent inducer of fibrosis. TGF-β1 has been extensively shown to play a vital role in promoting the production of collagen, which can result in kidney dysfunction during the fibrogenic phase of renal fibrosis (Roberts, 1998; Wang et al., 2005; Lan and Chung, 2011).

Short non-coding RNAs known as micro ribonucleic acids (miRNAs) inhibit the expression of specific genes via post-transcriptional repression of their respective target mRNAs. Notably, miRNAs have been increasingly shown to be key regulators of genes involved in the pathophysiology of fibrosis in multiple organs, including the kidney. Similarly, a growing body of evidence has demonstrated that the miR-29 family is the most fully characterized miRNA to be involved in TGF-β1-mediated fibrosis (van Rooij et al., 2008; Maurer et al., 2010; Cushing et al., 2011; Roderburg et al., 2011). Three highly similar orthologs, miR-29a, miR-29b, and miR-29c, compose the miR-29 family and share identical seed sequences (Xie et al., 2012). However, the various mechanisms that regulate kidney fibrosis and fibroblast activation have not yet been determined.

In our previous study, high-throughput sequencing and real-time PCR (RT-PCR) revealed the downregulation of miR-29c in UUO and 5/6 nephrectomy rat models (Xiaohan et al., 2012; Xie et al., 2012). Thus, the present study evaluates the mechanism by which miR-29c expression in fibroblasts regulates renal interstitial fibrosis.

## Methods Summary

### Unilateral Ureteral Obstruction

The surgical procedure was performed as previously described (Asada et al., 2011). Briefly, 6–12-week-old mice (acquired from the Model Animal Research Center, Nanjing University, China) were anesthetized and a mid-abdominal incision was made to expose the left ureter, after which a unilateral ureteral obstruction (UUO) was performed to obstruct the mid-ureter. A sham procedure, in which the left ureter was not obstructed, was applied to control mice. *In situ* perfusion with PBS and administration of 4% paraformaldehyde were performed before harvesting the kidneys, which were fixed in 4% paraformaldehyde and used for immunostaining. All experiments and methods involving live animals were performed after approval was received from the Institutional Animal Care and Use Committee of Wenzhou Medical University, China and in accordance with ethical guidelines for animal studies.

### Intra-Renal Pelvic Injection of rAAV6 Vectors

Recombinant adeno-associated virus (rAAV) vectors have great potential for clinical application. rAAV6 vector has been proven to have efficient renal transduction by direct intrarenal pelvis injection (Schreiber et al., 2016; Saito et al., 2019). In this study, rAAV6 carrying a FSP1 promoter that encodes miR-29c precursor (OE), miR-29c inhibitor (KD), β-catenin overexpression vector (AAV-β-catenin OE), β-catenin shRNA (AAV-shβ-catenin), TPM1 overexpression vector (AAV-TPM1 OE), or TPM1 shRNA (AAV-shTPM1), respectively, were all provided by OBiO Technology Corp. (Shanghai, China). The various rAAV vectors were introduced into isoflurane-anesthetized mice by intra-renal pelvic injection, and a flank incision was made to expose the left kidney of each animal. After locating the left ureter, the left renal pelvis was exposed. Using a 29-G needle, rAAV vectors (5 × 10^9^ genome copies/mouse) were gently injected into the left renal pelvis.

### Immunofluorescence

Paraffin sections of kidneys were subjected to immunofluorescence staining. After antigen retrieval, kidney sections were incubated overnight with anti-α-SMA (1:200, 19245; Cell Signaling Technology, Danvers, MA, United States) after permeabilization with 0.1% Triton X-100 in PBS and being blocked with 10% goat serum and 0.5% bovine serum albumin, followed by incubation with Alexa Fluor 488 conjugated secondary antibodies (1:500, 4416; Cell Signaling Technology). Cells were grown on glass coverslips for the purpose of cell staining. The samples were washed with PBS, fixed in 4% paraformaldehyde, and permeabilized with 0.5% Triton X-100 in PBS. Then, cells were stained with anti-beta-catenin (1:100, 8480; Cell Signaling Technology), followed by Alexa Fluor 488 conjugated secondary antibodies (1:500, 4416; Cell Signaling Technology). Slides were mounted with a medium containing DAPI (P0131; Beyotime, Shanghai, China). A Leica TCS SP8 confocal laser scanning microscope (Leica, Wetzlar, Germany) was used to capture images.

### Cell Culture

NRK-52E (rat renal tubular epithelial cells) were cultured in RPMI-1640 medium supplemented with 10% fetal bovine serum (FBS; Invitrogen, Carlsbad, CA, United States). NRK-49F (rat renal fibroblast cells) were cultured in DMEM-F12 supplemented with 10% fetal bovine serum (FBS; Invitrogen, Carlsbad, CA, United States). For the TGF-β1 treatment, NRK-49F cells were cultured (at 80% confluence) in a complete liquid medium with 10% FBS for 24 h, after which the cells were incubated in a serum-free medium for 16 h; then, 2 ng/mL recombined human TGF-β1 (R&D Systems, Minneapolis, MN, United States) were used to treat cells. miR-29c mimic or miR-29c inhibitor (Shanghai GenePharma, Shanghai, China) were transfected using lipofectamine 2000 (Invitrogen) based on the manufacturer’s protocol.

### Real-Time PCR

Trizol reagent (Invitrogen) was used to extract total RNA from cells as previously described (Xie et al., 2012). RNA quality was assessed using an Agilent 2100 Bioanalyzer (Agilent Technologies, Santa Clara, CA, United States). SYBR Green chemistry (Toyobo, Osaka, Japan) was used to assess mRNA levels of *Collagen 3* (*Col3a1*), *fibronectin*, *HGF*, and α*-SMA* as described previously (Xie et al., 2012). In the following analyses, total RNA samples were used.

### Western Blotting

After cells were lysed, electrophoresis of equal amounts of protein was conducted on a 12% SDS-polyacrylamide gel. Separated proteins were transferred to polyvinylidene fluoride membranes, which were then blocked for 1 h at room temperature using 5% skim milk in phosphate-buffered saline containing 0.1% Tween-20 (PBST). Then, the membranes were incubated overnight at 4°C with the following primary anti-bodies: TPM1 (1:1000, 3910; Cell Signaling Technology), α-SMA (1:250, ab7817; Abcam, Cambridge, United Kingdom), Col1a1 (1:1000, ab6308; Abcam), β-catenin (1:1000, 8480; Cell Signaling Technology), GSK3β (1:1000, 9315; Cell Signaling Technology), p-GSK3β (Ser9) (1:1000, ab131097; Abcam), and β-actin (1:1000, ab8224; Abcam). After the membranes were washed three times with PBST, they were incubated for 2 h at room temperature in either HRP-goat-anti-rabbit (ab6721, Abcam) or HRP-goat-anti-mouse (ab6789, Abcam) secondary antibodies. After membranes were washed with PBST three times, immunoreactive bands were visualized using Pierce ECL Plus western blotting substrate (32132; Thermo Fisher Scientific, Waltham, MA, United States) and quantified with Quantity One software (Bio-Rad Laboratories, Hercules, CA, United States). Lamin B1 and β-actin were used as internal loading controls.

### MicroRNA Target Prediction

To identify potential microRNA targets, the following microRNA databases and target prediction tools were used: miRNADA,^1^ microCosm,^2^ and miRDB,^3^ and miRNAMAP.^4^

### Luciferase Assay

TPM1-3′UTR-wild-type and TPM1-3′UTR-mutant-type 3′UTR plasmids were constructed using the psiCHECK-2 vector. The inserted sequence was 766 base pairs in length and contained the wild-type or mutant 3′UTR. For the luciferase reporter assay, plasmids and miR-29c were co-transfected into cells. Luciferase activities were measured 48 h after transfection using the Dual-Luciferase Reporter Assay System (Promega, Madison, WI, United States) and normalized to the negative control group.

### Statistical Analyses

Results are reported as mean ± SEM values, and differences between treatment means were assessed using unpaired 2-tailed Student’s *t*-tests for comparisons between two experimental groups and one-way ANOVA for comparisons between multiple groups using SPSS version 18 (SPSS Inc., Chicago, IL, United States). A *post hoc* Bonferroni test was used after ANOVA was conducted to assess significant differences between groups. A two-tailed *p*-value of less than 0.05 was used as the threshold for statistical significance. The latter two statistical analyses were conducted with GraphPad Prism (GraphPad Software, Inc., La Jolla, CA, United States).

## Results

### MiR-29c Is a Key Mediator of TGF-β1-Induced Renal Fibrosis in NRK-49F Cells

**FIGURE 1:**
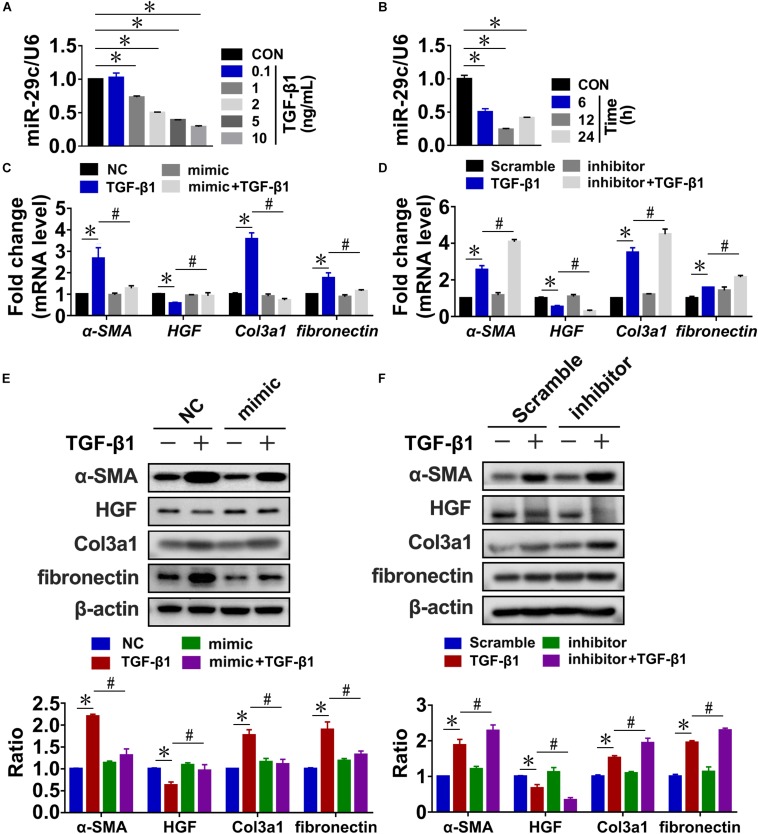
MiR-29c is an important mediator of TGF-β1-induced renal fibrosis in NRK-49F cells. RT-PCR results of miR-29c expression in NRK-49F cells treated with different dosages of TGF-β1 **(A)** or over different culture durations with TGF-β1 **(B)**. RT-PCR results of expression levels of several fibrosis-related genes (α-SMA, HGF, fibronectin, and Col3a1) in NRK-49F cells infected with miR-29c mimic **(C)** or miR-29c inhibitor **(D)** followed by 12 h of PBS or TGF-β1 (10 ng/mL) treatment. Representative western blots show levels of α-SMA, HGF, fibronectin, and Col3a1 proteins in NRK-49F cells infected with miR-29c mimic **(E)** or miR-29c inhibitor **(F)** followed by 12 h of PBS or TGF-β1 (10 ng/mL) treatment. Protein expression was normalized with β-actin. Data presented are mean ± SEM values. Symbols (“^∗^” and “#”) represent statistical significance.

In our previous studies, high-throughput sequencing and RT-PCR assays revealed that miR-29c levels were significantly downregulated in rat kidneys after UUO or 5/6 nephrectomy (Xiaohan et al., 2012; Xie et al., 2012). And we found that miR-29c exhibits higher expression in NRK-49F (rat renal fibroblast cells) than in NRK-52E (rat renal tubular epithelial cells) (Supplementary Figure S1A). To determine whether TGF-β1 increased the expression of miR-29c in fibrotic kidneys, we assayed miR-29c expression in TGF-β1-treated rat kidney fibroblasts (NRK-49F cells). RT-PCR confirmed that TGF-β1 treatment downregulated miR-29c expression in a dose- and time-dependent manner relative to the control, troughing at 12 h with an optimal dose of 10 ng/mL (Figures 1A,B). In contrast, the mRNA levels of fibrosis-related genes, including α*-SMA*, *fibronectin*, *and Col3a1*, uniformly increased based on the TGF-β1 treatment dosage and duration, accompanied by a decrease of *HGF* mRNA level. The protein levels of these factors showed consistent results (Supplementary Figure S1).

Next, we assessed the possible role of miR-29c in renal fibrosis *in vitro* by transient transfection of NRK-49F cells with miR-29c mimic or miR-29c inhibitor. In the miR-29c overexpression cells, there was a significant elevation of miR-29c expression, whereas the knockdown (KD) efficiency of miR-29c expression almost reached 80% (Supplementary FigureS S1G,H). As shown in Figures 1C,D, the mRNA levels of *HGF*, α*-SMA*, and *Col3a1* induced by TGF-β1 were markedly reversed by the miR-29c mimic treatment but enhanced by treatment with the miR-29c inhibitor. We also confirmed that miR-29c mimic attenuated the effects of TGF-β1. In contrast, miR-29c inhibitor further increased the protein levels of α-SMA, fibronectin, and Col3a1, and inhibited the protein level of HGF in TGF-β1-treated NRK-49F cells (Figures 1E,F).

Collectively, these results confirm the role of miR-29c in TGF-β1-mediated renal fibrosis, suggesting the potential for a therapeutic approach targeting miR-29c in kidney fibroblasts in order to suppress renal fibrosis in fibrotic kidneys.

### Therapeutic Effects of Targeting MiR-29c on Renal Fibrosis in Mice

**FIGURE 2:**
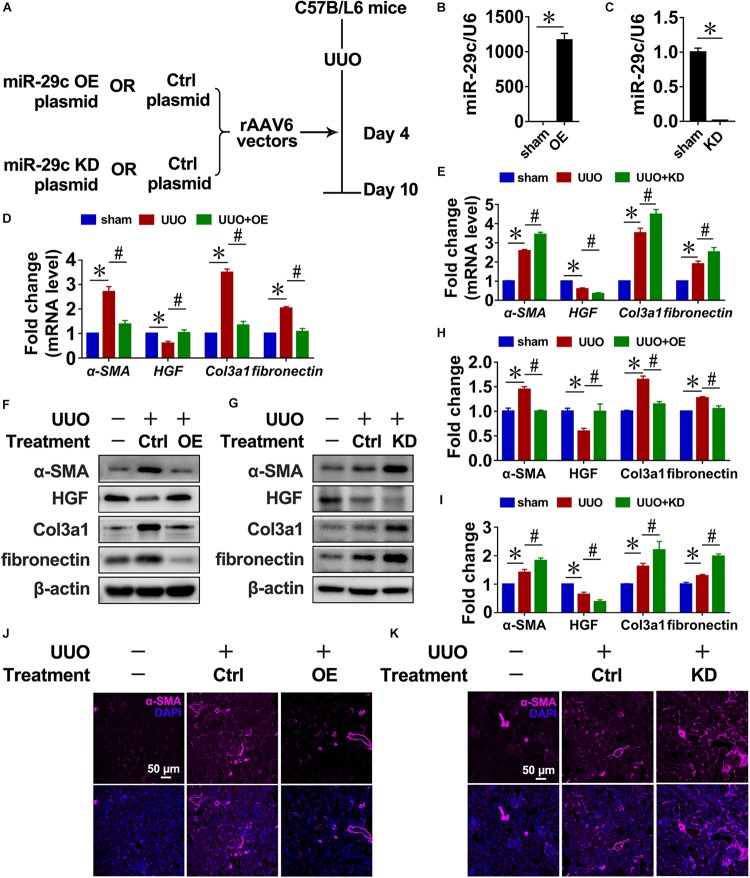
Targeting miR-29c has therapeutic effects on renal fibrosis in mice. **(A)** Schematic diagram of the experimental approach. RT-PCR results of miR-29c expression in kidneys after gene transfer of rAAV6 carrying pre-miR-29c (OE) **(B)** or miR-29c inhibitor (KD) **(C)**. RT-PCR results of the expression of several fibrosis-related genes (α-SMA, HGF, fibronectin, and Col3a1) in kidneys transfected with pre-miR-29c **(D)** or miR-29c inhibitor **(E)** 10 days after the sham procedure or UUO surgery. Representative western blots show levels of α-SMA, HGF, fibronectin, and Col3a1 proteins in kidneys transfected with pre-miR-29c **(F)** or miR-29c inhibitor **(G)** 10 days after the sham procedure or UUO surgery. Protein expression was normalized with β-actin. The data presented are mean ± SEM values. **(H)** Quantitative analysis of the protein levels in **(F)**. **(I)** Quantitative analysis of the protein levels in **(G)**. Representative photomicrographs of kidney sections transfected with pre-miR-29c **(J)** or miR-29c inhibitor **(K)** 10 days after the sham procedure or UUO surgery, stained for α-SMA, and counterstained with DAPI (blue). The data are presented as mean ± SEM values. Symbols (“^∗^” and “#”) represent statistical significance.

Next, we infected mice with recombinant adeno associated virus (rAAV) carrying a FSP1 promoter that encodes miR-29c precursor (pre-miR-29c) to examine the potential of miR-29c in renal fibrosis treatment (Figure 2A). Intense miR-29c signals were detected by RT-PCR in the renal fibroblasts of mice infected with rAAV6-Pre-miR-29c (Figure 2B). The ability of rAAV6-mediated miR-29c delivery to suppress renal fibrosis was next assessed in the UUO mouse model, revealing that rAAV6-mediated miR-29c gene transfer halted the loss of miR-29c from UUO kidneys by day 7. As confirmed by RT-PCR, western blot, and histological assays, this attenuated various markers of renal fibrosis progression were manifest in mRNA levels (Figure 2D), protein levels (Figure 2J), and α-SMA immunofluorescent staining (Figures 2F,H), respectively.

Mice were infected with rAAV carrying miR-29c inhibitor (anti-sense miR-29c) to confirm the effect of miR-29c deficiency on renal fibrosis (Figures 2A,C). Thus, miR-29c knockdown in UUO mouse kidneys significantly increased tubulointerstitial fibrosis severity, as assessed via α-SMA immunofluorescent staining (Figure 2K). Additionally, the negative effects of miR-29c deficiency on mRNA and protein markers of fibrosis were further validated by the RT-PCR and western blot (Figures 2E,G,I).

Thus, miR-29c overexpression had a therapeutic effect on renal disease through inhibition of renal fibrosis progression.

### TGF-β1-Induced Expression of MiR-29c Is Mediated by Wnt/β-Catenin Signaling *in vivo* and *in vitro*

The accumulation of myofibroblasts, which originate from resident pericytes and fibroblasts, underlies renal fibrosis progression (Humphreys et al., 2010; Asada et al., 2011; Kramann et al., 2013; Duffield, 2014; Mack and Yanagita, 2015). As TGF-β1 is a fibrogenesis-propagating and -inducing cytokine that can regulate miR-29c expression, the signaling mechanisms through which TGF-β1 regulates the expression of miR-29c in kidney fibroblasts were investigated further. It is well documented that the robust activation of Wnt signaling is induced by UUO; moreover, Wnt antagonists have been shown to ameliorate renal fibrosis and repress myofibroblast activation in CKD models (Surendran et al., 2005; He et al., 2009; Hao et al., 2011; Zhou et al., 2013).

Treatment of NRK-49F cells with ICG-001, an established Wnt/β-catenin antagonist, significantly prevented the effects induced by TGF-β1 of several fibrosis-related genes (α*-SMA*, *HGF*, *Col3a1*, *and fibronectin*) and miR-29c (Supplementary Figures S2A,C,E,F), suggesting that Wnt/β-catenin activation may be necessary for the observed TGF-β1-induced inhibition of miR-29c expression. Additionally, NRK-49F treated with CHIR-98014 (an activator of Wnt signaling) further inhibited miR-29c expression in response to TGF-β1 (Supplementary Figure S2B). Also, the detrimental effects of TGF-β1-induced downregulation of miR-29c were associated with significant α-SMA, Col3a1, and fibronectin upregulation and HGF downregulation in NRK-49F cells, which were enhanced in β-catenin-overexpressing NRK-49F cells (Supplementary Figures S2D,G,H).

**FIGURE 3:**
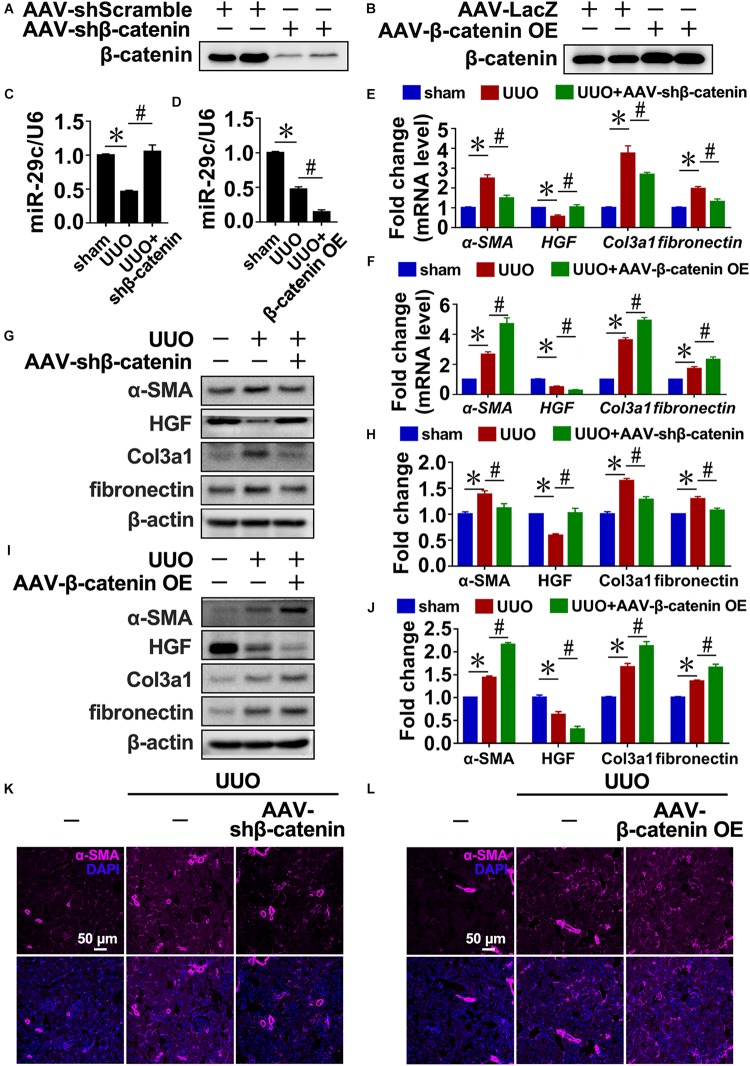
TGF-β1 inhibited miR-29c expression via Wnt/β-catenin signaling *in vivo.* Western blot results of β-catenin expression in kidneys after transfection with AAV-shβ-catenin **(A)** or AAV-β-catenin OE constructs **(B)**. Protein expression was normalized with β-actin. RT-PCR results of miR-29c expression in kidneys after transfection with AAV-shβ-catenin **(C)** or AAV-β-catenin OE constructs **(D)**. RT-PCR results assessing the expression of several fibrosis-related genes (α-SMA, HGF, fibronectin, and Col3a1) in kidneys transfected with AAV-shβ-catenin **(E)** or AAV-β-catenin OE constructed **(F)** 10 days after the sham procedure or UUO surgery. Representative western blots show protein levels of α-SMA, HGF, fibronectin, and Col3a1 in kidneys transfected with AAV-shβ-catenin **(G)** or AAV-β-catenin OE constructs **(I)** 10 days after the sham procedure or UUO surgery. Protein expression was normalized with β-actin. **(H)** Quantitative analysis of the protein levels in **(G)**. **(J)** Quantitative analysis of the protein levels in **(I)**. Representative photomicrographs of the kidney sections transfected with AAV-shβ-catenin **(K)** or AAV-β-catenin OE constructs **(L)** 10 days after the sham procedure or UUO surgery, stained for α-SMA, and counterstained with DAPI (blue). The data are presented as mean ± SEM values. Symbols (“^∗^”, “#”, “&” and “^∧^”) represent statistical significance.

Similarly, the disruption of β-catenin in UUO kidneys increased miR-29c expression (Figures 3A,C) and attenuated interstitial fibrosis progression compared with the UUO mouse kidneys (Figures 3E,G,H,K). In contrast, delivery of β-catenin overexpression plasmids via rAAV demonstrated that miR-29c expression was further inhibited in UUO kidneys (Figures 3B,D) and accompanied by aggravated kidney fibrosis (Figures 3L). Also, several fibrotic markers exhibited enhanced mRNA and protein levels (Figures 3F,I,J), confirming the regulatory role of Wnt/β-catenin in miR-29c expression in response to TGF-β1 in fibrosis.

### Reciprocal Regulation of MiR-29c and Wnt/β-Catenin Pathway in Renal Fibroblasts

We have confirmed that the expression of HGF, a known Wnt/β-catenin-targeted gene (Zhou et al., 2018), was restored after overexpression of miR-29c in TGF-β1-treated NRK-49F cells and UUO mouse kidneys. As a potent anti-fibrotic factor, HGF both antagonizes the action of TGF-β1 and represses renal inflammation (Liu, 2004; Giannopoulou et al., 2008). Therefore, we analyzed the potential involvement of miR-29c in modulating renal fibrosis through the Wnt/β-catenin pathway.

**FIGURE 4:**
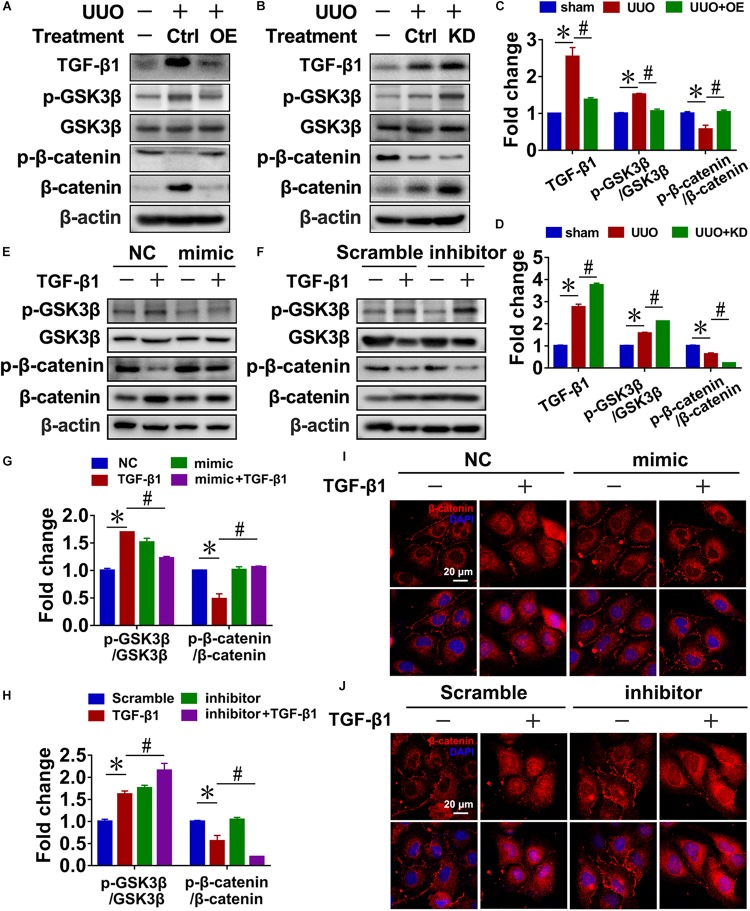
Reciprocal regulation of miR-29c and the Wnt/β-catenin pathway in renal fibroblasts. Representative western blots show levels of TGF-β1, p-GSK3β, GSK3β, p-β-catenin, and β-catenin proteins in kidneys transfected with pre-miR-29c **(A)** or miR-29c inhibitor **(B)** 10 days after the sham procedure or UUO surgery. Protein expression was normalized with β-actin. **(C)** Quantitative analysis of the protein levels in **(A)**. **(D)** Quantitative analysis of the protein levels in **(B)**. Representative western blots show protein levels of TGF-β1, p-GSK3β, GSK3β, p-β-catenin, and β-catenin in NRK-49F cells infected with miR-29c mimic **(E)** or miR-29c inhibitor **(F)** followed by a 12-h PBS or TGF-β1 (10 ng/mL) treatment. **(G)** Quantitative analysis of the protein levels in **(E)**. **(H)** Quantitative analysis of the protein levels in **(F)**. Representative photomicrographs of NRK-49F infected with miR-29c mimic **(I)** or miR-29c inhibitor **(J)** followed by a 12-h PBS or TGF-β1 (10 ng/mL) treatment, staining for β-catenin, and counterstaining with DAPI (blue). The data are presented as mean ± SEM values. Symbols (“^∗^” and “#”) represent statistical significance.

In UUO mice, pre-miR-29c significantly inhibited the activation of Wnt/β-catenin signaling, as demonstrated by decreased levels of p-GSK3β/GSK3β and increased levels of p-β-catenin/β-catenin, and prevented TGF-β1 upregulation (Figures 4A,C). In contrast, we found that miR-29c knockdown in mice further accelerated Wnt/β-catenin pathway activation in UUO mice (Figures 4B,D).

Next, we investigated the effect of miR-29c on Wnt/β-catenin signaling in NRK-49F cells, finding that miR-29c mimic in TGF-β1-treated NRK-49F cells significantly inhibited the activation of Wnt/β-catenin signaling, as demonstrated by decreased p-GSK3β/GSK3β levels, increased p-β-catenin/β-catenin levels, and decreased nuclear localization of β-catenin (Figures 4E,G,I) compared with the TGF-β1-treated NRK-49F cells. Additionally, loss of miR-29c in NRK-49F further exacerbated Wnt/β-catenin activation (Figures 4F,H,J).

These results collectively indicate that miR-29c is likely involved in controlling the Wnt/β-catenin pathway and thus renal fibrosis. Accordingly, the reciprocal regulation of miR-29c action and the Wnt/β-catenin pathway may have a critical role in the production of ECM in renal fibroblasts.

### Tpm1 is a Potential MiR-29c Target During Renal Fibrosis

**FIGURE 5:**
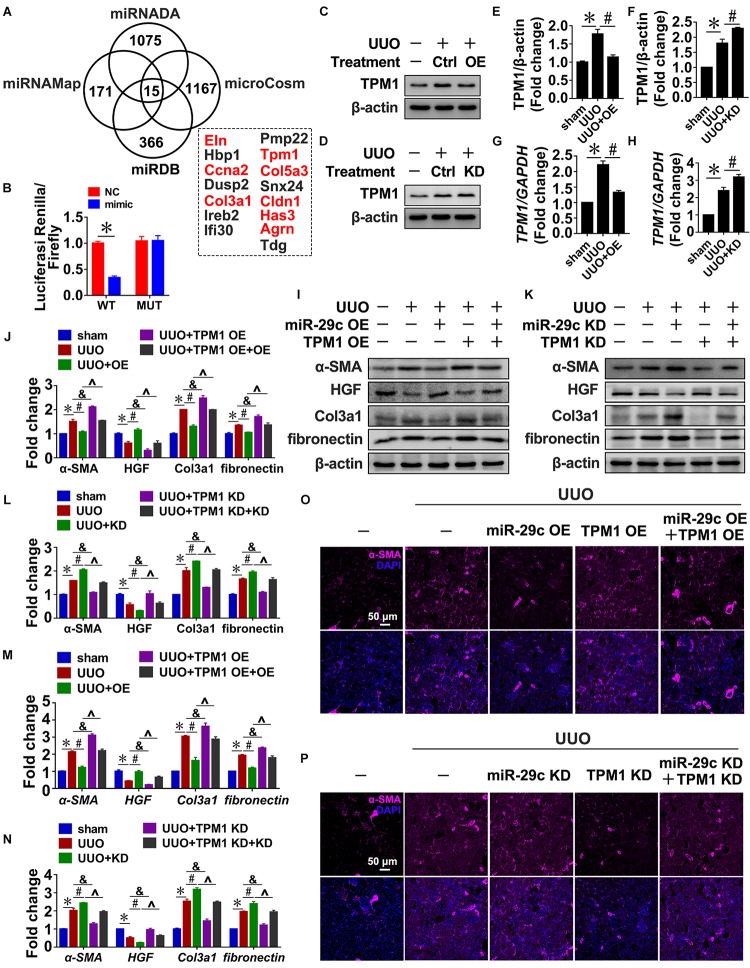
Tpm1 is a potential miR-29c target during renal fibrosis. **(A)** Schematic diagram summarizing target genes identified using four microRNA target prediction programs (miRNADA, microCosm, miRDB, and miRNAMAP) with the potential targets identified by all four of the programs. **(B)** Luciferase reporter activity assay performed in HEK 293T cells infected with a WT reporter plasmid (WT) + miR-29c or a mutant 3’UTR TPM1 reporter plasmid (MUT) + miR-29c. Representative western blots show protein levels of TPM1 in kidneys transfected with pre-miR-29c **(C)** or miR-29c inhibitor **(D)** 10 days after the sham procedure or UUO surgery. Protein expression was normalized with β-actin. **(E)** Quantitative analysis of the protein levels in **(C)**. **(F)** Quantitative analysis of the protein levels in **(D)**. RT-PCR results of TPM1 expression in kidneys transfected with pre-miR-29c **(G)** or miR-29c inhibitor **(H)** 10 days after the sham procedure or UUO surgery. Representative western blots show levels of α-SMA, HGF, fibronectin, and Col3a1 proteins in kidneys transfected with pre-miR-29c + AAV-TPM1 OE **(I)** or miR-29c inhibitor + AAV-shTPM1 construct **(K)** 10 days after the sham procedure or UUO surgery. Protein expression was normalized with β-actin. **(J)** Quantitative analysis of the protein levels in **(I)**. **(L)** Quantitative analysis of the protein levels in **(K)**. RT-PCR results for the expression of several fibrosis-related genes (α-SMA, HGF, fibronectin, and Col3a1) in kidneys transfected with pre-miR-29c + AAV-TPM1 OE **(M)** or miR-29c inhibitor + AAV-shTPM1 construct **(N)** 10 days after the sham procedure or UUO surgery. Representative photomicrographs of the kidney sections transfected with pre-miR-29c + AAV-TPM1 OE **(O)** or miR-29c inhibitor + AAV-shTPM1 construct **(P)** 10 days after the sham procedure or UUO surgery, stained for α-SMA, and counterstained with DAPI (blue). The data are presented as mean ± SEM values. Symbols (“^∗^”, “#”, “&” and “^∧^”) represent statistical significance.

To identify potential miR-29c targets, four microRNA target prediction programs (miRNADA, microCosm, miRNAMAP, and TargetScan) were used. Because each program produced different predictions of microRNA targets due to their differing algorithms, the only targets considered were those predicted by all four programs, which was done in order to minimize false positive results. As a result, we identified eight potential targets associated with fibrosis: Eln, Ccna2, Col3a1, Tpm1, Col5a3, Cldn1, Has3, and Agrn (Figure 5A). TPM1 was selected for further study because TGF-β1 treatment of NRK-49F cells indeed upregulated TPM1 expression in a time- and dose-dependent manner (Supplementary Figure S3A,B), and TPM1 has recently been reported to play a role in stress fiber function (Pittenger et al., 1994; Coutu et al., 2004; Houle et al., 2007; Morita et al., 2007). Thus, 3’UTR-mediated interactions between miR-29c and TPM1 were directly assessed using a 3′UTR reporter analysis. Compared with a control miRNA mimic, the miR-29c mimic significantly reduced TPM1 3′UTR activity, while it had no significant effect on mutated TPM1 3′UTRs (Figure 5B).

In addition, miR-29c mimic significantly decreased TPM1 expression in NRK-49F cells, while miR-29c inhibitor further aggravated TGF-β1-induced upregulation of TPM1, as could be observed by increases in both protein and mRNA levels (Supplementary Figures S3C–H). Also, the observed negative association was further corroborated by the UUO mouse results (Figures 5C–H), indicating that TPM1 expression was negatively associated with expression of miR-29c and renal fibrosis occurrence.

Next, we assessed the functional contributions of TPM1 on miR6-29c in renal fibrosis. Expression levels of α-SMA, fibronectin, and Col3a1 expression induced by TGF-β1 were significantly inhibited by TPM1 deficiency but enhanced by TPM1 overexpression in both *in vivo* and *in vitro* models. In addition, the decreased protein levels of HGF induced by TGF-β1 were restored by TPM1 deficiency but accelerated by TPM1 overexpression (Figures 5I–N and Supplementary Figures S3I–N). TPM1 overexpression partially reversed miR-29c overexpression-induced anti-fibrotic effects (Figure 5O), as reflected by the significantly elevated protein and mRNA levels of α-SMA, Col3a1, fibronectin, and decreased protein and mRNA levels of HGF (Figures 5I,J,M). Additionally, in TPM1-deficient mice, miR-29c deficiency-induced upregulation of several fibrotic genes was abolished (Figures 5K,L,N), and ameliorate renal fibrosis (Figure 5P). We were able to consistently confirm the effects of TPM in NRK-49F cells. These results collectively indicate that TPM1 is necessary in the regulatory role of miR-29c in TGF-β1-induced renal fibrosis. Finally, we investigated the relationship between TPM1 and β-catenin. As shown in Supplementary Figure S4, the disruption or overexpression of β-catenin can affect the expression levels of TPM1 in renal fibroblasts (Supplementary Figure S4A–F), while TPM1 can activate the Wnt/β-catenin pathway (Supplementary Figures S4G–L), which further confirm the reciprocal regulation of miR-29c and Wnt/β-catenin pathway in renal fibroblasts.

## Discussion

In this study, we found that miR-29c expression was downregulated in UUO mouse kidneys as well as TGF-β1-treated NRK-49F cells, which thus inhibits myofibroblast formation via targeting of TPM1. Additionally, the production of ECM in renal fibroblasts appears to be controlled by the reciprocal regulation of miR-29c action and the Wnt/β-catenin pathway.

Fibroblasts are the key cells that drive renal fibrosis, because they are able to produce substantial amounts of ECM, a major component of renal fibrosis (Hinz, 2010). Blocking the transdifferentiation of renal fibroblasts to myofibroblast is critical to blocking renal fibrosis. Previously, miR-29 members have been shown to be downstream targets of TGF-β1 (Qin et al., 2011; Wang et al., 2012). Both experimental and bioinformatical analyses have also demonstrated the role of miR-29s as anti-fibrotic factors through the direct targeting of many ECM genes (Sengupta et al., 2008; Li et al., 2009; Kriegel et al., 2012). In our previous studies, we found that the expression of miR-29c, but not miR-29a or miR-29b, significantly decreased with renal interstitial fibrosis progression. Here, we show that miR-29c may be an essential regulator of TGF-β1 signaling involved in renal fibrosis. In UUO model and in TGF-β1-treated NRK-49F cells, miR-29c function gain or loss evidently modulated TGF-β1-mediated fibrosis. Low delivery efficiencies of chemically synthesized microRNAs during the *in vivo* experiment led us to use an AAV-mediated delivery strategy to modulate miR-29c expression in mice, which may offer novel insights for future kidney gene therapy approaches.

Through the targeted inhibition of Wnt/β-catenin signaling, kidney fibrotic lesions can be ameliorated in pre-clinical settings. Although quiescent in normal adult kidneys, Wnt/β-catenin signaling is observed across a wide variety of fibrotic CKD cases (Tan et al., 2014; Gewin, 2018). DKK1, Klotho, sFRP4, β-catenin inhibitor ICG-001, and other exogenous Wnt antagonists can be delivered to significantly reduce renal β-catenin accumulation and thus inhibit expression of its various target genes. Wnt antagonists have also been shown to ameliorate renal fibrosis and repress myofibroblast activation in CKD models (Surendran et al., 2005; He et al., 2009; Hao et al., 2011; Zhou et al., 2013). The present study supports the hypothesis that Wnt/β-catenin inhibition by ICG-001 can prevent TGF-β1-induced miR-29c downregulation and ameliorate existing kidney damage.

Additionally, the Wnt/β-catenin pathway in kidney fibroblasts may be controlled by miR-29c expression through a positive feedback loop. Overexpression of miR-29c by rAAV6-Pre-miR-29c in mice effectively inhibited the activation of Wnt/β-catenin signaling in fibrotic kidneys, suggesting that the reciprocal regulation observed between Wnt/β-catenin signaling and miR-29c in renal fibroblasts may play a key role in fibrogenic activation. At present, the mechanism by which miR-29c downregulates TGF-β1 remains unclear. As miR-29c action is associated with HGF, a potent anti-fibrotic factor that antagonizes TGF-β1 activity (Giannopoulou et al., 2008; Zhou et al., 2018), repression of TGF-β1 by miR-29c may be mediated by HGF; more research is required to test this hypothesis.

A search of target prediction databases for putative miR-29c targets led to the discovery of TPM1 as a potential miR-29c target. TPM1 belongs to the high molecular weight group of tropomyosins. A series of studies have demonstrated that TPM1 acts as stress fibers that are responsible for epithelial mesenchymal transformation and ECM deposition, and plays a key role in the occurrence and development of myocardia fibrosis (Pittenger et al., 1994; Coutu et al., 2004; Houle et al., 2007; Morita et al., 2007). This information links TPM1 with the Wnt/β-catenin pathway. The results of this study show that TPM1 is a miR-29c target during renal fibrosis, and reciprocal expression of miR-29c and TPM1 has been found in both NRK-49F cells and mouse kidneys. Additionally, TPM1 exhibited the ability to regulate both TGF-β1-induced fibrosis and the Wnt/β-catenin pathway, indicating that targeting TPM1 by miR-29c may have a critical role in UUO-induced renal fibrosis.

## Conclusion

**FIGURE 6:**
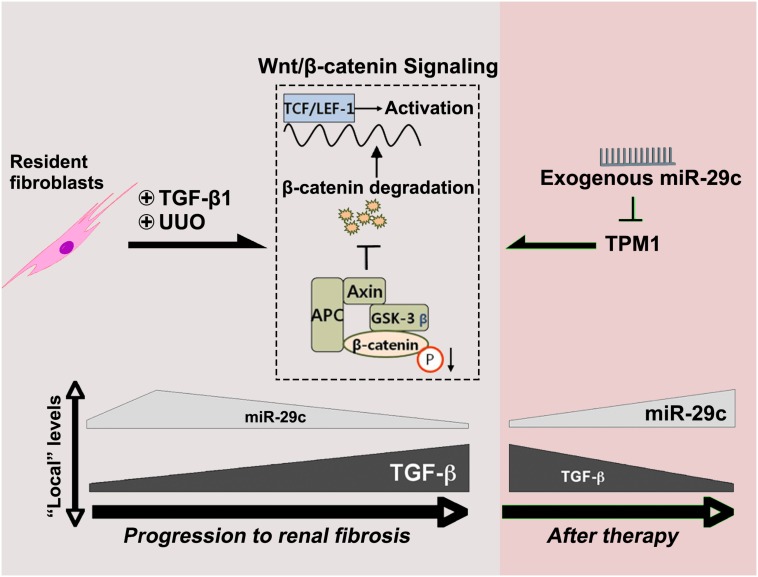
MiR-29c alleviates renal fibrosis via TPM1-mediated suppression of the Wnt/β-catenin pathway. In UUO model and in TGF-β1-treated NRK-49F cells, miR-29c expression is inhibited by TGFβ1 via the Wnt/β-catenin signaling. Meanwhile, miR-29c is able to inhibit the Wnt/β-catenin pathway by suppressing TPM1 expression.

In the present study, TGF-β1 was demonstrated to regulate miR-29c expression through Wnt/β-catenin signaling. In contrast, miR-29c appears to inhibit the Wnt/β-catenin pathway by suppressing TPM1 expression (Figure 6). As suggested by this feedback mechanism, miR-29c may be a key fibrosis-related microRNA expressed by fibroblasts in TGF-β1/Wnt/β-catenin-driven renal fibrosis, and manipulation of miR-29c action may accordingly offer a potential therapeutic pathway for renal fibrosis treatment.

## Data Availability Statement

The datasets generated for this study are available on request to the corresponding author.

## Ethics Statement

The animal study was reviewed and approved by the Institutional Animal Care and Use Committee of Wenzhou Medical University, China.

## Author Contributions

All authors listed have made a substantial, direct and intellectual contribution to the work, and approved it for publication.

## Conflict of Interest

The authors declare that the research was conducted in the absence of any commercial or financial relationships that could be construed as a potential conflict of interest.

## Footnotes

1 http://www.microrna.org/microrna/home.do

2 http://www.ebi.ac.uk/enright-srv/microcosm

3 http://mirdb.org/

4 http://mirnamap.mbc.nctu.edu.tw/

## Supplementary Material

FIGURE S1 TGF-β1-induced renal fibrosis in NRK-49F cells. **(A)** RT-PCR results for miR-29c expression in NRK-52E and NRK-49F cells. RT-PCR results for the expression of several fibrosis-related genes (α-SMA, HGF, fibronectin, and Col3a1) in NRK-49F cells treated with different dosages of TGF-β1 **(B)** or treated for different culture durations with TGF-β1 **(C)**. Representative western blots show levels of α-SMA, HGF, fibronectin, and Col3a1 proteins in NRK-49F cells treated with different dosages of TGF-β1 **(D)** or for different culture durations with TGF-β1 **(F)**. **(E)** Quantitative analysis of the protein levels in **(D)**. **(G)** Quantitative analysis of the protein levels in **(F)**. RT-PCR results for miR-29c expression in NRK-49F cells infected with miR-29c mimic **(H)** or miR-29c inhibitor **(I)**. The data are presented as mean ± SEM values. Symbol (“^∗^”) represents statistical significance.

FIGURE S2 TGF-β1 inhibited miR-29c expression via Wnt/β-catenin signaling *in vitro.* RT-PCR results of miR-29c expression in NRK-49F cells treated with ICG-001 **(A)** or CHIR-98014 **(B)**. RT-PCR results for the expression of several fibrosis-related genes (α-SMA, HGF, fibronectin, and Col3a1) in NRK-49F cells treated with ICG-001 **(C)** or CHIR-98014 **(D)**. Representative western blots show levels of α-SMA, HGF, fibronectin, and Col3a1 proteins in NRK-49F cells treated with ICG-001 **(E)** or CHIR-98014 **(G)**. Protein expression was normalized with β-actin. **(F)** Quantitative analysis of the protein levels in **(E)**. **(H)** Quantitative analysis of the protein levels in **(G)**. The data are presented as mean ± SEM values. Symbols (“^∗^” and “#”) represent statistical significance.

FIGURE S3 Tpm1 is a potential target of miR-29c during renal fibrosis. Representative western blots show TPM1 protein levels in NRK-49F cells treated with different dosages of TGF-β1 **(A)** or for different culture durations with TGF-β1 **(B)**. Representative western blots show TPM1 protein levels in NRK-49F cells infected with miR-29c mimic **(C)** or miR-29c inhibitor **(D)** followed by a 12-h PBS or TGF-β1 (10 ng/mL) treatment. **(E)** Quantitative analysis of the protein levels in **(C)**. **(F)** Quantitative analysis of the protein levels in **(D)**. RT-PCR results of TPM1 expression in NRK-49F cells infected with miR-29c mimic **(G)** or miR-29c inhibitor **(H)** followed by a 12-h PBS or TGF-β1 (10 ng/mL) treatment. Representative western blots show protein levels of α-SMA, HGF, fibronectin, and Col3a1 in NRK-49F cells infected with miR-29c mimic+Ad-TPM1 OE **(I)** or miR-29c inhibitor+Ad-shTPM1 **(K)** followed by a 12-h PBS or TGF-β1 (10 ng/mL) treatment. **(J)** Quantitative analysis of the protein levels in **(I)**. **(L)** Quantitative analysis of the protein levels in **(K)**. RT-PCR results for the expression of several fibrosis-related genes (α-SMA, HGF, fibronectin, and Col3a1) in NRK-49F cells infected with miR-29c mimic+Ad-TPM1 OE (M) or miR-29c inhibitor+Ad-shTPM1 (N) followed by a 12-h PBS or TGF-β1 (10 ng/mL) treatment. The data are presented as mean ± SEM values. Symbols (“^∗^”, “#”, “&” and “^∧^”) represent statistical significance.

FIGURE S4 Tpm1 is a potential miR-29c target during renal fibrosis. Western blot results of TPM1 expression in kidneys after transfection with AAV-shβ-catenin **(A)** or AAV-β-catenin OE constructs **(B)** 10 days after the UUO surgery. Protein expression was normalized with β-actin. Western blot results of TPM1 expression in NRK-49F cells treated with ICG-001 **(C)** or CHIR-98014 **(D)** followed by a 12-h TGF-β1 (10 ng/mL) treatment. Protein expression was normalized with β-actin. **(E)** RT-PCR results of TPM1 expression in kidneys after transfection with AAV-shβ-catenin or AAV-β-catenin OE constructs. **(F)** RT-PCR results of TPM1 expression in NRK-49F cells treated with ICG-001 or CHIR-98014. Representative photomicrographs of NRK-49F infected with miR-29c mimic **(G)** or miR-29c inhibitor **(H)** followed by a 12-h TGF-β1 (10 ng/mL) treatment, staining for β-catenin (red), and counterstaining with DAPI (blue). Representative western blots and quantitative analysis of protein levels in kidneys transfected with AAV-TPM1 OE or AAV-TPM1 OE + pre-miR-29c **(I)**; AAV-shTPM1 or AAV-shTPM1 + miR-29c inhibitor **(J)** 10 days after the UUO surgery. Protein expression was normalized with β-actin. Representative western blots and quantitative analysis of protein levels in NRK-49F cells infected with Ad-TPM1 OE or Ad-TPM1 OE + miR-29c mimic **(K)**; Ad-shTPM1 or Ad-shTPM1 + miR-29c inhibitor **(L)** followed by a 12-h TGF-β1 (10 ng/mL) treatment. The data are presented as mean ± SEM values. Symbols (“^∗^” and “#”) represent statistical significance.
