# Supplementary figures and images for: The MicroRNA MiR-29c Alleviates Renal Fibrosis via TPM1-Mediated Suppression of the Wnt/β-Catenin Pathway
Source: Front Physiol. 2020 Apr 14;11:331. doi: 10.3389/fphys.2020.00331 (PMC7171049; doi:10.3389/fphys.2020.00331)

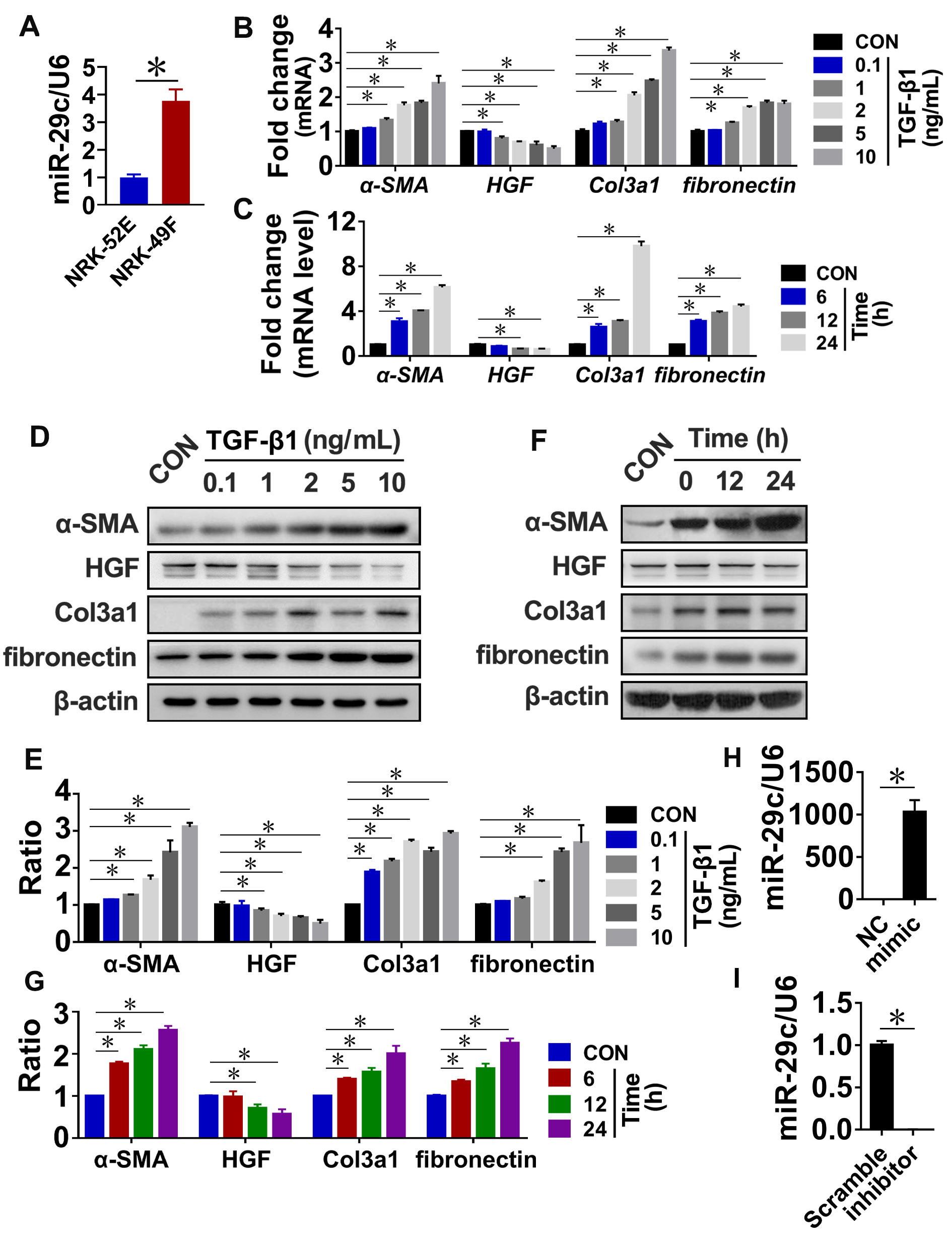

Supplement: FIGURE S1 — TGF-β1-induced renal fibrosis in NRK-49F cells. (A) RT-PCR results for miR-29c expression in NRK-52E and NRK-49F cells. RT-PCR results for the expression of several fibrosis-related genes (α-SMA, HGF, fibronectin, and Col3a1) in NRK-49F cells treated with different dosages of TGF-β1 (B) or treated for different culture durations with TGF-β1 (C). Representative western blots show levels of α-SMA, HGF, fibronectin, and Col3a1 proteins in NRK-49F cells treated with different dosages of TGF-β1 (D) or for different culture durations with TGF-β1 (F). (E) Quantitative analysis of the protein levels in (D). (G) Quantitative analysis of the protein levels in (F). RT-PCR results for miR-29c expression in NRK-49F cells infected with miR-29c mimic (H) or miR-29c inhibitor (I). The data are presented as mean ± SEM values. Symbol (“∗”) represents statistical significance. [file Image_1.TIF]

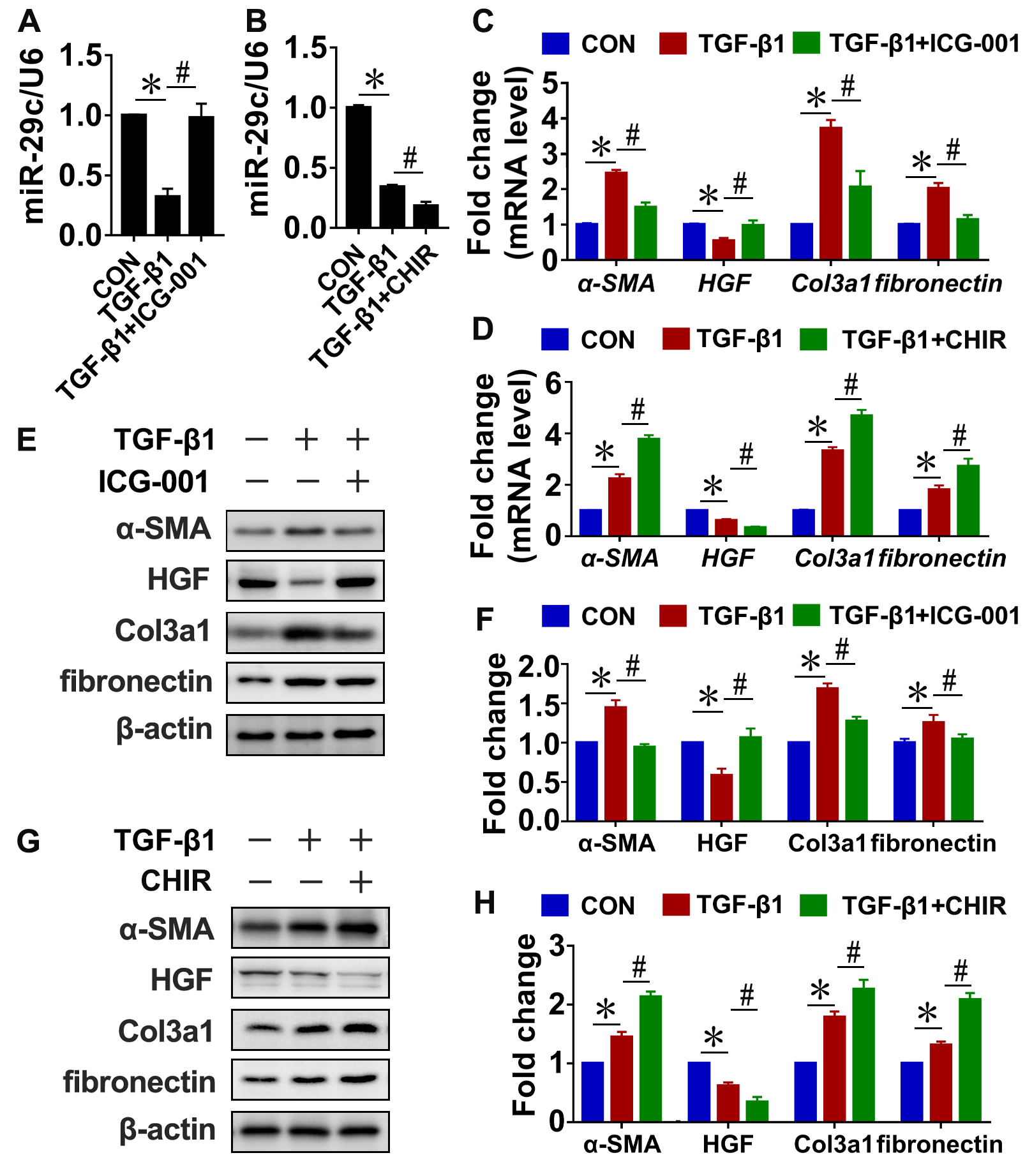

Supplement: FIGURE S2 — TGF-β1 inhibited miR-29c expression via Wnt/β-catenin signaling in vitro. RT-PCR results of miR-29c expression in NRK-49F cells treated with ICG-001 (A) or CHIR-98014 (B). RT-PCR results for the expression of several fibrosis-related genes (α-SMA, HGF, fibronectin, and Col3a1) in NRK-49F cells treated with ICG-001 (C) or CHIR-98014 (D). Representative western blots show levels of α-SMA, HGF, fibronectin, and Col3a1 proteins in NRK-49F cells treated with ICG-001 (E) or CHIR-98014 (G). Protein expression was normalized with β-actin. (F) Quantitative analysis of the protein levels in (E). (H) Quantitative analysis of the protein levels in (G). The data are presented as mean ± SEM values. Symbols (“∗” and “#”) represent statistical significance. [file Image_2.TIF]

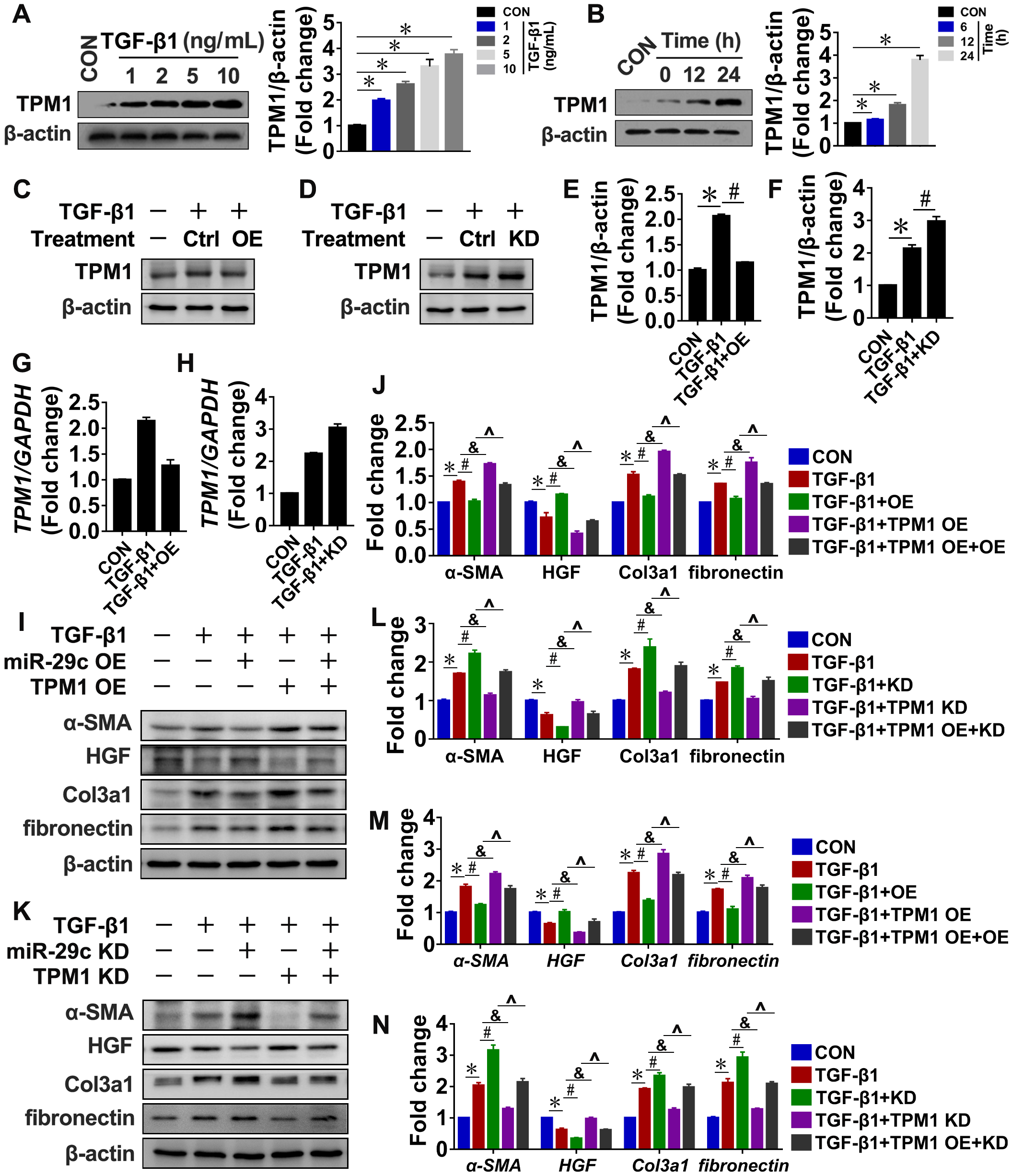

Supplement: FIGURE S3 — Tpm1 is a potential target of miR-29c during renal fibrosis. Representative western blots show TPM1 protein levels in NRK-49F cells treated with different dosages of TGF-β1 (A) or for different culture durations with TGF-β1 (B). Representative western blots show TPM1 protein levels in NRK-49F cells infected with miR-29c mimic (C) or miR-29c inhibitor (D) followed by a 12-h PBS or TGF-β1 (10 ng/mL) treatment. (E) Quantitative analysis of the protein levels in (C). (F) Quantitative analysis of the protein levels in (D). RT-PCR results of TPM1 expression in NRK-49F cells infected with miR-29c mimic (G) or miR-29c inhibitor (H) followed by a 12-h PBS or TGF-β1 (10 ng/mL) treatment. Representative western blots show protein levels of α-SMA, HGF, fibronectin, and Col3a1 in NRK-49F cells infected with miR-29c mimic+Ad-TPM1 OE (I) or miR-29c inhibitor+Ad-shTPM1 (K) followed by a 12-h PBS or TGF-β1 (10 ng/mL) treatment. (J) Quantitative analysis of the protein levels in (I). (L) Quantitative analysis of the protein levels in (K). RT-PCR results for the expression of several fibrosis-related genes (α-SMA, HGF, fibronectin, and Col3a1) in NRK-49F cells infected with miR-29c mimic+Ad-TPM1 OE (M) or miR-29c inhibitor+Ad-shTPM1 (N) followed by a 12-h PBS or TGF-β1 (10 ng/mL) treatment. The data are presented as mean ± SEM values. Symbols (“∗”, “#”, “&” and “∧”) represent statistical significance. [file Image_3.TIF]

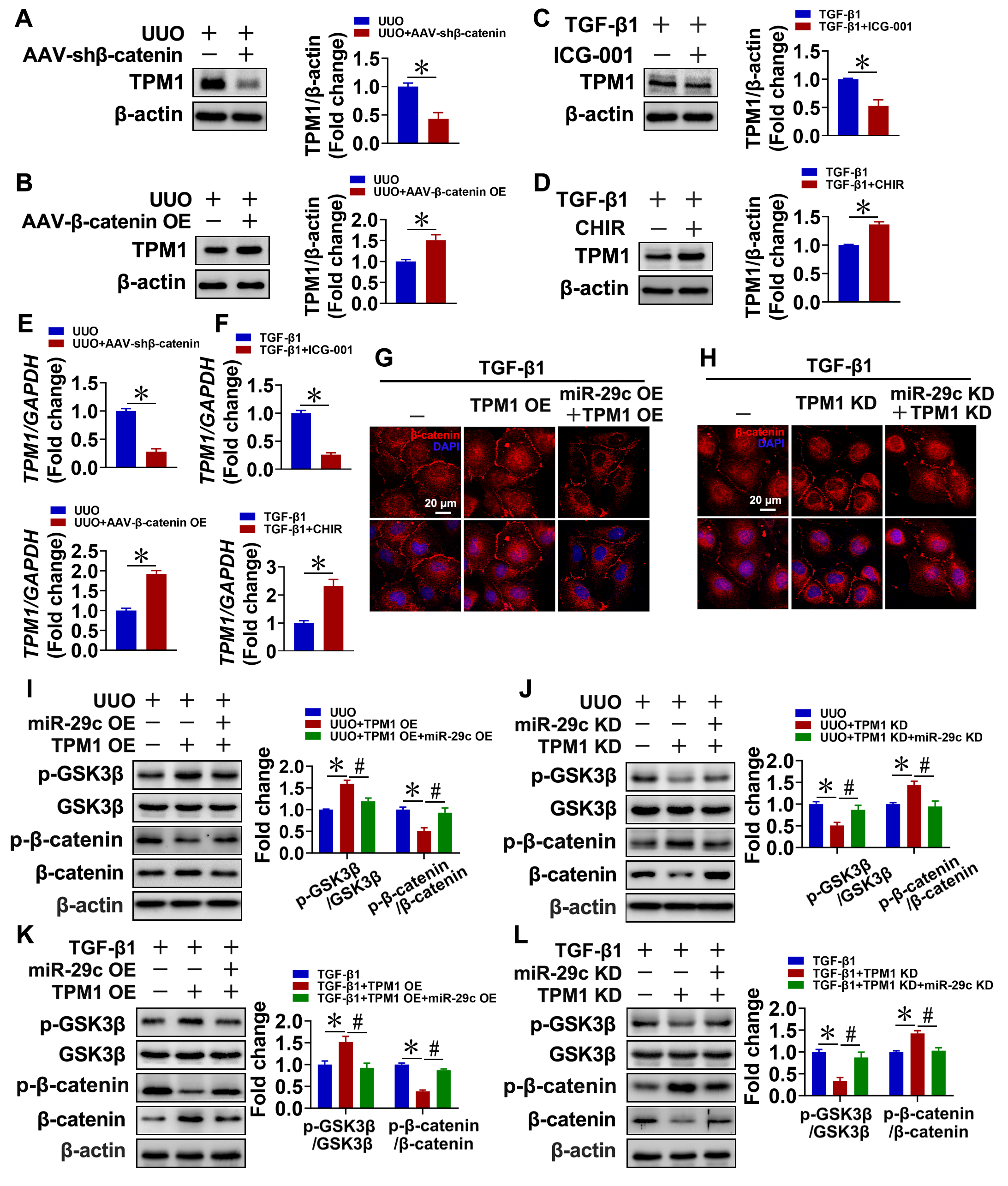

Supplement: FIGURE S4 — Tpm1 is a potential miR-29c target during renal fibrosis. Western blot results of TPM1 expression in kidneys after transfection with AAV-shβ-catenin (A) or AAV-β-catenin OE constructs (B) 10 days after the UUO surgery. Protein expression was normalized with β-actin. Western blot results of TPM1 expression in NRK-49F cells treated with ICG-001 (C) or CHIR-98014 (D) followed by a 12-h TGF-β1 (10 ng/mL) treatment. Protein expression was normalized with β-actin. (E) RT-PCR results of TPM1 expression in kidneys after transfection with AAV-shβ-catenin or AAV-β-catenin OE constructs. (F) RT-PCR results of TPM1 expression in NRK-49F cells treated with ICG-001 or CHIR-98014. Representative photomicrographs of NRK-49F infected with miR-29c mimic (G) or miR-29c inhibitor (H) followed by a 12-h TGF-β1 (10 ng/mL) treatment, staining for β-catenin (red), and counterstaining with DAPI (blue). Representative western blots and quantitative analysis of protein levels in kidneys transfected with AAV-TPM1 OE or AAV-TPM1 OE + pre-miR-29c (I); AAV-shTPM1 or AAV-shTPM1 + miR-29c inhibitor (J) 10 days after the UUO surgery. Protein expression was normalized with β-actin. Representative western blots and quantitative analysis of protein levels in NRK-49F cells infected with Ad-TPM1 OE or Ad-TPM1 OE + miR-29c mimic (K); Ad-shTPM1 or Ad-shTPM1 + miR-29c inhibitor (L) followed by a 12-h TGF-β1 (10 ng/mL) treatment. The data are presented as mean ± SEM values. Symbols (“∗” and “#”) represent statistical significance. [file Image_4.TIF]
